# Extracellular Matrix Proteome and Phosphoproteome of Potato Reveals Functionally Distinct and Diverse Canonical and Non-Canonical Proteoforms

**DOI:** 10.3390/proteomes4030020

**Published:** 2016-06-24

**Authors:** Eman Elagamey, Kanika Narula, Arunima Sinha, Pooja Rani Aggarwal, Sudip Ghosh, Niranjan Chakraborty, Subhra Chakraborty

**Affiliations:** National Institute of Plant Genome Research, Aruna Asaf Ali Marg, New Delhi 110067, India; schakraborty@nipgr.ac.in (E.E.); gayatrinarula@gmail.com (K.N.); arunima35@gmail.com (A.S.); aggarwal.pooja28@yahoo.com (P.R.A.); sgblackpearl@gmail.com (S.G.); nchakraborty@nipgr.ac.in (N.C.)

**Keywords:** ECM, proteoforms, phosphoproteome, potato, 2-DE, protein interaction network

## Abstract

The extracellular matrix (ECM) has a molecular machinery composed of diverse proteins and proteoforms that combine properties of tensile strength with extensibility exhibiting growth-regulatory functions and self- and non-self-recognition. The identification of ECM proteoforms is the prerequisite towards a comprehensive understanding of biological functions accomplished by the outermost layer of the cell. Regulatory mechanisms of protein functions rely on post-translational modifications, phosphorylation in particular, affecting enzymatic activity, interaction, localization and stability. To investigate the ECM proteoforms, we have isolated the cell wall proteome and phosphoproteome of a tuberous crop, potato (*Solanum tuberosum*). LC-MS/MS analysis led to the identification of 38 proteins and 35 phosphoproteins of known and unknown functions. The findings may provide a better understanding of biochemical machinery and the integrated protein and phosphoprotein network of ECM for future functional studies of different developmental pathways and guidance cues in mechanosensing and integrity signaling.

## 1. Introduction

Cellular homeostasis, semantics, and mechanical attributes are complex processes involving recruitment of cellular fibrils and differentiation of extracellular matrix (ECM). The extracellular matrix exhibits a prominent feature of combining extreme tensile strength with extensibility. Plasticity of ECM is a prerequisite for the expansion and elongation of the cell by helping reorganize cell wall networks and remodel cellular machinery. Cellular growth depends on the balance between mechanical force and turgor pressure mechanics. This represents a nexus of various biological processes linking growth and development, plant-pathogen interactions, abiotic stress, self- and inter-organismal recognition, signaling components and metabolic processes. ECM integrates various internal and external cues, notably nutritional, stress factors, and auto-regulatory signals. Composition and structure of ECM varies across plant kingdom. Cell morphogenesis is governed by the functional integrity and maintenance mechanism in the ECM [1]. Therefore, assembly and disassembly of ECM components or modulation of signal transduction pathways in terms of biological activity is a common theme.

ECM hydration, deposition, extension, cross-linking and mechanosensing are accurately regulated in a temporal, spatial, and developmental manner at translational and post-translational levels. The multifunctional feature of ECM houses a compendium of dynamic, heterogeneous, and complex proteins and phosphoproteins. ECM proteins comprise less than 10% of cell wall dry weight [2] and represent a comprehensive catalog of the cell wall proteins that spread throughout the extracellular milieu and range from highly mobile with no apparent interaction, to covalently-bound proteins. ECM protein populations of many plant organs and tissues have been reported [3,4,5,6,7] together with detailed methodologies to optimize extraction [8,9,10]. In fact, different ECM proteins, particularly phosphoprotein epitopes, are not evenly distributed (or accessible) within the cell wall, nor are these epitopes distributed uniformly across two adjacent walls [11]. Reversible and sub-stoichiometric protein phosphorylation is an important posttranslational modification for cell-cell communication and signal transduction in cellular processes. However, phosphorylation studies of ECM proteins in plants have not been investigated to date. Phosphoproteomics that examines phosphorylation changes may help in determining ECM signaling regulation at system level.

Potato (*Solanum tuberosum* L.) is the fourth most important temperate food crop consumed worldwide and has about 200 wild species of various ploidy levels. Of which, tetraploids are the most commonly cultivated species of potato all over the world. It is an excellent example of a vegetatively-propagated plant species with complex genetics/genome [12]. There are many unanswered questions of how cell wall dynamics are regulated and coordinated with other cellular processes in potato. Proteoform subsets in different organelles fulfil discrete, but varied, cellular functions and provide additional important information about protein localization and pathway compartmentalization. Thus, understanding ECM proteoforms might play major role in unraveling translational and post-translational information that modulates the biological processes and molecular functions in the outermost layer in potato.

Here, we performed a preliminary proteomic and phosphoproteomic analysis of the extracellular matrix of tetraploid potato using two-dimensional gel electrophoresis (2-DE) in combination with LC-MS/MS analyses. Functional cataloguing of proteins and phosphoproteins from purified potato ECM fraction defined translational activities and architectural reorganization dynamics. This study presents the first ECM proteome and phosphoproteome report in a tuber crop. Furthermore, protein and phosphoprotein network highlights that wall-associated signaling components, wall hydration, extension, deposition, and mechonosensing are the major functions of ECM in plant cells.

## 2. Experimental Section

### 2.1. Plant Material and Growth Condition

Potato (*Solanum tuberosum* L.) cultivar Kufri Chipsona-1 was grown and subcultured on Murashige and Skoog MS media [13] by taking seven node explants to get a uniform size of seedlings in an environmentally-controlled growth room maintained at 25 °C ± 2 °C, 50% ± 5% relative humidity, under a 16 h photoperiod (270 μmol m^−2^ s^−1^ light intensity). The three-week-old seedlings were sampled as experimental materials, harvested, frozen in liquid nitrogen and stored at −80 °C.

### 2.2. Isolation of Pure ECM

The ECM fraction was isolated as described by Averyhart-Fullard et al., and Feiz et al. with few modifications [8,14]. Further, the ECM fraction was purified as described earlier [15]. In brief, 5.0 g of tissues were ground to powder in liquid nitrogen with 0.3% (*w*/*w*) polyvinylpolypyrollidone (PVPP) and transferred to 50 mL centrifuge tube. Immediately, tissue powder was homogenized in low ionic strength homogenizing buffer (5 mM K_3_PO_4_, pH 6.0, 5 mM DTT, 1 mM PMSF) for 2 min to preserve the ionic bonds and to dilute the ionic strength of the ECM. The ECM fraction was recovered by differential centrifugation at 1000× *g* for 5 min at 4 °C. The pellet thus obtained was washed ten times with excess deionized water.

### 2.3. Light Microscopy

The purified ECM fraction was visualized using light microscopy as described in Bessire et al. [16]. In brief, the purified ECM fraction was fixed with Karnovsky’s fixative (2% (*v*/*v*) paraformaldehyde and 2.5% (*v*/*v*) glutaraldehyde) overnight at 4 °C. It was then washed in 0.1 M phosphate buffer pH 7.4 and treated with 1% osmium tetroxide. The fraction was dehydrated sequentially in acetone and embedded in epoxy embedding resin (45359 1EA-F; Sigma Aldrich, St. Louis, MO, USA). Thin sections were made and stained with toluidine blue and examined by a Nikon Eclipse 80i microscope (Nikon, Minato, Tokyo, Japan)

### 2.4. ECM Protein Extraction and Quantification

The ECM fraction was suspended in three volumes (*w*/*v*) of extraction buffer (200 mM CaCl_2_, 5 mM DTT, 1 mM PMSF, 0.3% (*w*/*w*) PVPP) and extracted on a shaking platform for 2 h at 4 °C to remove any further contaminants and to release ECM proteins. Proteins were separated from the insoluble ECM fraction by centrifugation (10,000× *g*) for 10 min at 4 °C and filtered through a 0.45 μm filter. The filtrate was concentrated using a Centricon YM3 (Millipore, Bedford, MA, USA) and then dialyzed overnight against 1000 volumes of deionized water with one change. The concentration of protein extract was determined by Bradford assay (Bio-Rad, Hercules, CA, USA).

### 2.5. Isolation of Plasma Membrane Proteins

Extraction of plasma membrane fraction was carried out according to Santoni et al. [17]. Fifteen g of three-week-old seedlings were homogenized in homogenization buffer (50 mM sucrose, 50 mM tris, 10% (*w*/*v*) glycerol, 20 mM Na_2_-EDTA, 20 mM EGTA, 50 mM NaF, 5 mM β-glycerophosphate, 1 mM phenanthroline, 0.6% (*w*/*v*) PVP, and 10 mM ascorbic acid). The homogenate was centrifuged at 2500× *g* for 20 min. After filtering through Miracloth, the supernatant was ultracentrifuged at 1500× *g* for 30 min. The resultant microsomal pellet was resuspended in resuspension medium (5 mM phosphate buffer (pH 7.8), 330 mM sucrose, 2 mM DTT and 10 mM NaF). The microsomal fraction was distributed in 27 g phase systems containing 6.4% dextran, 6.4% PEG (3350), 5 mM phosphate buffer (pH 7.8), 2.5 mM KCl and 300 mM sucrose. The plasma membrane-enriched fractions were retrieved from the upper phase and diluted in washing buffer (10 mM Tris, 10 mM boric acid, 300 mM sucrose, 9 mM KCl, 5 mM Na_2_-EDTA, 5 mM EGTA, and 50 mM NaF). The suspension was centrifuged at 3100× *g* for 45 min at 4 °C to obtain the fraction.

### 2.6. Isolation of Cytosolic Proteins

Cytosolic proteins were extracted from 1 g of tissue in lysis buffer containing 26 mM Tris-HCl, 2 M thiourea, 0.3% (*v*/*v*) Triton X-100, 20 mM Trizma base, 7.5 M urea, 63 mM CHAPS, and the protease inhibitor cocktail complete mini (Roche, Mannheim, Germany). After shaking for 30 min at 4 °C, 7 mM of DTT was added to the extract. The protein extracts were stirred for 20 min and centrifuged twice at 20,000× *g* for 20 min at 4 °C. The final supernatant was precipitated in one volume of acetone containing 20% TCA, 9 mM DTT at −20 °C overnight, then centrifuged at 20,000× *g* for 20 min at 4 °C. The pellet was resuspended in two volumes of acetone containing 9 mM DTT at −20 °C for 1 h, then centrifuged as above to obtain the cytosolic fraction [18].

### 2.7. Enzyme Assay

Catalase and vanadate-inhibited H^+^ ATPase activities were assayed as described [15]. For catalase assay, the reaction mixture was prepared using 10 μg each of ECM and cytosolic proteins by adding 50 μL of protein extract to 940 μL of 70 mM potassium phosphate buffer (pH 7.5). Reaction was started by addition of 25 mM H_2_O_2_ and a decrease in absorbance at 240 nm was monitored for 5 min. Baseline correction was done by subtracting the absorbance taken without addition of H_2_O_2_. The assay was performed in triplicates and the absorbance values obtained were plotted against time. The vanadate-inhibited H^+^ ATPase was assayed with 20 μg of protein for ECM and plasma membrane fraction in 120 μL of assay buffer. The assay was performed in the presence and absence of 0.1 mM orthovanadate, freshly prepared and boiled in buffer prior to addition of 0.05% Triton X-100. The assay was performed in the presence of a detergent to expose all hidden sites of the cell wall bound plasma membrane, if any. The reaction was initiated by addition of ATP and was incubated at 37 °C for 30 min. Blanks lacking MgSO_4_ were run in parallel [19] and the released inorganic phosphate was determined [20]. The relative activity of the enzyme was calculated by taking the difference between the absorbance at 700 nm in absence and presence of orthovanadate.

### 2.8. Two-Dimensional Polyacrylamide Gel Electrophoresis

The ECM proteins were diluted in dilution buffer (100 mM Tris-Cl (pH 8.5), 20% (*v*/*v*) glycerol, 4% (*w*/*v*) SDS, 20 mM DTT and 1 mM PMSF) and boiled for 5 min. The protein sample was allowed to cool to room temperature (25 °C), precipitated with nine volumes of 100% chilled acetone overnight at −20 °C. The precipitates were recovered by centrifugation at 10,000× *g* at 4 °C, for 10 min. Protein pellets were washed twice with 80% acetone to remove excess SDS, air-dried and dissolved in 2-D rehydration buffer. Rehydration was carried out for 16 h with 300 μg protein resuspended in 200 μL rehydration buffer and 1000 μg protein resuspended in 450 μL rehydration buffer for 13 cm and 24 cm IPG strips, respectively. The rehydrated proteins were first separated by isoelectric focusing (IEF) using gel strips to form an immobilized pH gradient (Immobiline DryStrip, pH 4–7, 13 cm and pH 4–7, 24 cm; GE Healthcare, Chalfont St Giles, UK) and then by SDS-PAGE using 12.5% polyacrylamide gels. Isoelectric focusing was performed using IPGphor system (Amersham Biosciences, Bucks, UK) at 20 °C up to 30,000 Vhr for 13 cm and 80,000 Vhr for 24 cm IPG strips. The focused strips were reduced with 1% (*w*/*v*) DTT in 15 mL of equilibration buffer (6 M urea, 50 mM Tris-HCl (pH 8.8), 30% (*v*/*v*) glycerol and 2% (*w*/*v*) SDS) for 30 min, followed by alkylation with 2.5% (*w*/*v*) iodoacetamide in the same buffer for 30 min. For 2-D SDS PAGE, the strips were placed on top of 12.5% (*w*/*v*) acrylamide gels along with low range unstained markers (Bio-Rad) at basic end and PeppermintStick^TM^ Phosphoprotein markers (Invitrogen, Carlsbad, CA, USA) at acidic end. The electrophoresed proteins were stained to visualize phosphoproteins and total ECM proteins sequentially.

### 2.9. Detection of ECM Phosphoproteins and Proteins

In order to detect the phosphoproteins, 2-D gels were fixed overnight in fixation solution (50% methanol, 10% glacial acetic acid) and washed with deionized water three times each for 30 min on shaking. The gels were stained with Pro-Q Diamond phosphoprotein gel stain solution (Invitrogen, Carlsbad, CA, USA) for 2 h on an orbital shaker and destained using destaining solution (20% acetonitrile, sodium acetate (pH 4) (Sigma-Aldrich, St. Louis, MO, USA), three times each for 30 min) followed by washing with deionized water three times each for 10 min. After taking the image, the same gels were then stained with a silver stain plus kit (Bio-Rad) to detect total ECM proteins.

### 2.10. Image Acquisition and in Silico Analysis

After 2-DE and gel staining, the Pro-Q Diamond stained gels were scanned using a Typhoon scanner 9210 (GE Healthcare, Chalfont St Giles, UK) with an emission wavelength 580 nm and silver stained gels were scanned by a FluorS system equipped with a 12-bit camera (Bio-Rad). At least three 2-DE gels representing three biological replicates were included in the data analysis. The scanned gel images were processed and analyzed with PDQuest gel analysis software version 7.2.0 (Bio-Rad). All images were subsequently processed using the software settings for spot detection, background subtraction, spot quantitation, and gel-to-gel matching of spot patterns as described previously [21]. Each spot included in the standard gel met the criteria of being qualitatively consistent in size and shape in the replicate gels and being within the linear range of detection. A few landmarks in the gel series were manually defined to improve the automated matching results. In addition, for quantification (based on spot density and area), the PDQuest software was used to assign quality scores to each gel spot. Low-quality spots (quality score < 30) were removed before further analysis. Spots with a quality score greater than 30 that met the above-mentioned stringent criteria were thereafter referred to as high-quality spots. The high-quality spots were used to calculate the median value for a given spot and this value was used as the spot quantity on the standard gel. The correlation coefficient was maintained at a minimum of 0.8 between gel images for a high level of reproducibility (Figure S1). Next, for comparison, the protein spots were normalized to the “total density in gel image” mode and spots were manually annotated. Experimental molecular mass and *p*I were calculated from digitized 2-DE images using standard molecular mass marker proteins.

### 2.11. Peptide Preparation for Tandem Mass-Spectrometry

Protein spots were mechanically excised from silver-stained gels and in-gel digested with trypsin, and peptides were extracted according to standard techniques [22]. In brief, the excised gel plugs were washed twice with deionized water and destained using 100 mM sodium thiosulfate and 30 mM potassium ferricyanide solution (1:1 *v*/*v*) for 15 min with shaking. The gel pieces were washed twice in 25 mM ammonium bicarbonate in 50% ACN with gentle shaking for 10 min. The gel pieces were then dehydrated by soaking in 100% acid-free ACN for 5 min and vacuum dried for 20 to 30 min. Tryptic digestion was performed by incubating the gel pieces in 10 μL of trypsin (10 ng/μL) overnight at 30 °C. The dried gel pieces were digested with 0.2 ng/μL cold trypsin (Sigma-Aldrich, St. Louis, MO, USA) prepared in 25 mM ammonium bicarbonate in ultrapure water. Subsequently, the gel pieces were incubated on ice for 10 min to allow gel pieces to swell and absorb the trypsin solution, then 25 μL of 25 mM ammonium bicarbonate in ultrapure water were added to cover gel pieces. Samples were incubated at 30 °C overnight with shaking. The extracted peptides were collected in a fresh tube. To further extract peptides, 10 μL of 1% (*v*/*v*) trifluoroacetic acid solution were added to each gel piece and incubated for 5 min. Extraction solution was removed and added to digestion mixture.

### 2.12. Protein Identification Using LC-MS/MS

For LC-MS/MS analysis, trypsin-digested eluted peptides were loaded onto a C18 Pep-Map100 column (3 mm, 100 Å, 75 mm ID_15 cm; LCPackings (Dionex, Thermo Fisher Scientific, Waltham, MA, USA)) at 300 nL min^−1^, using a linear gradient of water/acetonitrile/0.1% formic acid (*v*/*v*) and analyzed by electrospray ionization using the Ultimate 3000 Nano HPLC system (Dionex, Thermo Fisher Scientific) coupled to a 4000 QTRAP mass spectrometer (Applied Biosystems/MDS SCIEX, Foster City, CA, USA). The peptides were eluted with a gradient of 10% to 40% acetonitrile with 0.1% formic acid (*v*/*v*) over 60 min. The MS/MS data were extracted using Analyst software version 1.5.1 (Applied Biosystems). Peptide analysis was performed through data-dependent acquisition of mass spectrometry scans (mass-to-charge ratio from 400 to 1800), followed by MS/MS scans. Peptides were identified by searching the peak list against the Viridiplantae (Green Plants) (4,026,576 sequences) with NCBInr database (8,994,603 sequences; 307,8807,967 residues) using the Mascot version 2.1 search engine (www.matrixscience.com, Matrix Science, Boston, MA, USA).

The database search criteria were as follows: taxonomy, all entries; peptide tolerance, ±1.2 D; MS/MS tolerance, ±0.6 D; peptide charge, +1, +2, or +3; maximum allowed missed cleavage, 1; fixed modification, Cys carbamidomethylation; variable modification, Met oxidation; and instrument type, electrospray ionization (ESI)-ion trap. The score threshold to achieve *p* < 0.05 was set by the Mascot algorithm and was based on the size of the database used in the search. We considered only those protein spots whose molecular weight search score was above the significant threshold level determined by Mascot. For the number of observed peptides per protein, the unique sequences were counted and were imported to Excel spreadsheets (Table S1). For proteins identified by only one peptide having a score higher than 40 the peptide sequence was systematically checked manually (Table S2).

### 2.13. Network Visualization

Protein-protein interactions (PPI) were searched against geneMANNIA [23], BAR [24], STRING [25], menthe [26], Interoporc [27], IntAct [28], DIP [29], APID [30], MINT [31], and BIND [32] PPI databases and the in silico generated protein and phosphoprotein network was visualized with Cytoscape [33] version 3.0.2 [34].

### 2.14. Bioinformatic Analysis

The protein functions were assigned using protein function database Pfam or InterPro or through literature survey. Since the functional annotation was based on Pfam and InterPro, the functional redundancy was, thus, greatly minimized. The identified proteins were divided into different functional classes according to gene ontology (GO) and literature. The BLASTP search of identified protein sequences was performed through Blast2GO [35] against the Uniprot protein database with a minimum expectation value of 1 × 10^−3^. Annotations were retrieved with default parameters: pre-eValue-Hit-Filter at 1 × 10^−6^, cut-off was set at 55 and GO weight at 5. To understand the function of unknown proteins, we performed domain analysis to predict the conserved domain(s) using the InterProScan [36] database. The subcellular localization of the putative phosphoproteins was predicted using TargetP [37], WolfPSORT [38] and YLoc+ [39] programs. NetPhos 2.0 [40] analysis was performed to predict the putative site(s) of phosphorylation.

## 3. Results

### 3.1. Evaluation of Potato ECM Integrity and Purity Assessment

**Figure 1:**
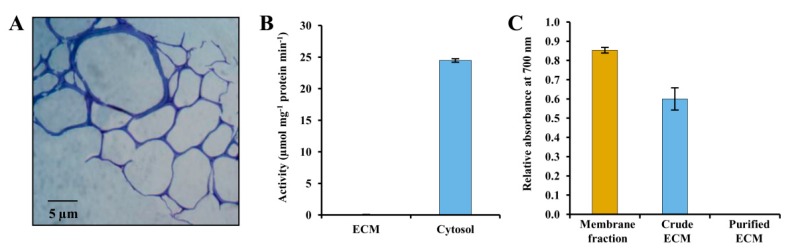
Analysis of isolated potato ECM fraction and determination of its purity. (**A**) Micrograph of a toluidine blue-stained section of potato leaflet showing the ECM specificity of the staining. (**B**) Determination of catalase specific activity in potato ECM and cytosolic fraction. The cytosolic fraction prepared from potato tissue for catalase activity was used as a positive control. (**C**) Determination of vanadate-inhibited H^+^ ATPase activity in the potato ECM and plasma membrane fraction. The plasma membrane fraction of potato was used as a positive control. Data represents experiments performed in triplicate and indicated as ± SD.

To construct a 2-DE map of the potato ECM proteoforms, we isolated the ECM protein fraction by mechanical disruption and fractionation processes. The ECM-enriched pellet was washed repeatedly and extracted with low-osmolarity buffer to remove any contaminants from other organelles. The complete cell breakage was confirmed by analysis of isolated ECM fraction using a stereo zoom microscope. The micrographs showed that the ECM fraction was free from plasma membrane or other ultrastructural cytoplasmic organelles and that there were no intact cells which had escaped breakage during homogenization (Figure 1A). The purity of the ECM was evaluated further by assaying organelle-specific marker enzyme activities that could possibly contaminate the preparation. Catalase activity was assessed for peroxisome, while vanadate-inhibited H^+^ ATPase activity was determined for the plasma membrane contamination [19]. The crude cell extracts (ECM pellet before washing) showed high catalase and ATPase activities, whereas the ECM fraction did not show any significant activities of both the enzymes (Figure 1B,C). These results altogether suggest that cytosolic and plasma membrane contaminations of the ECM, if any, were beyond detectable limits. Thus, in our study, the purity of the ECM was carefully considered for further analyses.

### 3.2. Construction of 2-DE ECM Proteome and Phosphoproteome Map

**Figure 2:**
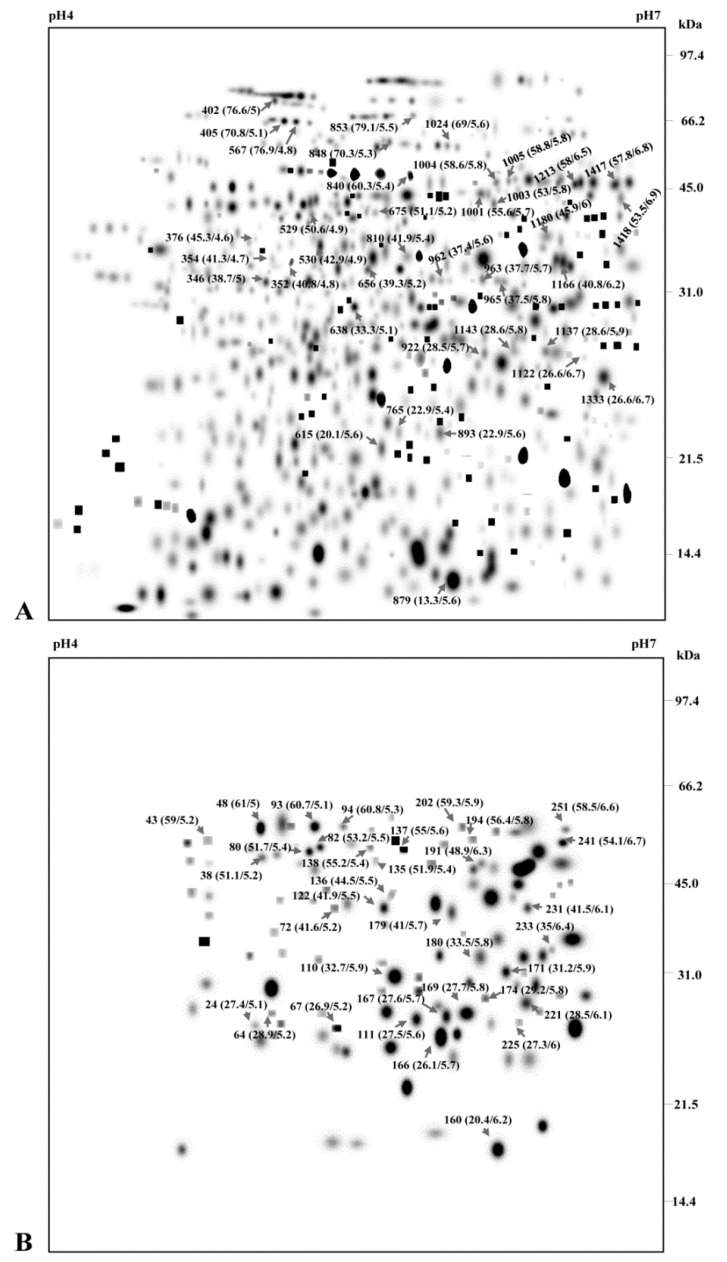
ECM proteome and phosphoproteome profiles. (**A**) ECM proteome profile of silver stained 2-DE gels. (**B**) ECM phosphoproteome profile of Pro-Q stained 2-DE gels. The experiments were carried out in triplicates and further computationally combined into a representative standard gel. Each spot is indicated by red arrow and accompanied with spot number bracketed with Mw and p*I*.

ECM proteins and phosphoproteins were separated by 2-DE to establish a reference map. Initially, the ECM proteome and phosphoproteome was resolved onto a 13 cm pH 3–10 that revealed more than 250 spots. However, proteins in the basic range exhibited poor resolution (Figure S2A). To make the reference map well resolved, we used 13 cm narrow pH range (pH 4–7) that showed distribution of most of the protein spots in the p*I* range of 4–7 and the molecular mass range of 14 to 100 kDa with vertical streaking (Figure S2B,C). The reference map was made more comprehensive and vertical streaking was reduced by using 24 cm IPG strips of p*I* range of 4–7 as the number of spots increased with strip length and the resolution of spots was enhanced using 24 cm gels (Figure 2).

More than 329 protein spots and 88 phosphoprotein spots were detected on 13 cm gels p*I* 4–7, out of which 300 protein spots and 79 phosphoprotein spots survived the filtering process. While 24 cm pH 4–7 strips showed 733 protein spots and 259 phosphoprotein spots, with 704 high-quality protein spots and 241 phosphoprotein spots matched to our filtering criteria. The spots were numbered as StEP-1 to StEP-733 and StEPP-1 to StEPP-259 (the letteres ‘St’ identify the organism (*Solanum tuberosum*) and ‘E’ signifies the subcellular organelle (ECM) from which the proteome ‘P’ and phosphoproteome ‘PP’ map has been made, whereas the numerals indicate the spot numbers (Figure 2A,B).

### 3.3. Distribution Pattern and Physicochemical Characteristics of ECM Proteins and Phosphoproteins

A total of 38high-quality protein spots and 35 phosphoprotein spots were excised and identified by ion trap LC-MS/MS Figure S3, Table 1 and Table 2). Venn diagram showed that four identified proteins and phosphoproteins were common between the two datasets (Figure S4). On the basis of the average quantitative expression data, ECM proteins and phosphoproteins were categorized into three major classes: high abundance (35.0%; >1%–2% of the total protein load, and 67.74%; >1%–2% of the total phosphoprotein load, and medium abundance (20.6%; 0.5%–1% of the total protein load, and 17.74%; 0.5%–1% of the total phosphoprotein load). Low abundant proteins account for 44.6% (0.03%–0.5% of the total protein load) and 14.5% (0.03%–0.5% for the phosphoprotein load).

**Figure 3:**
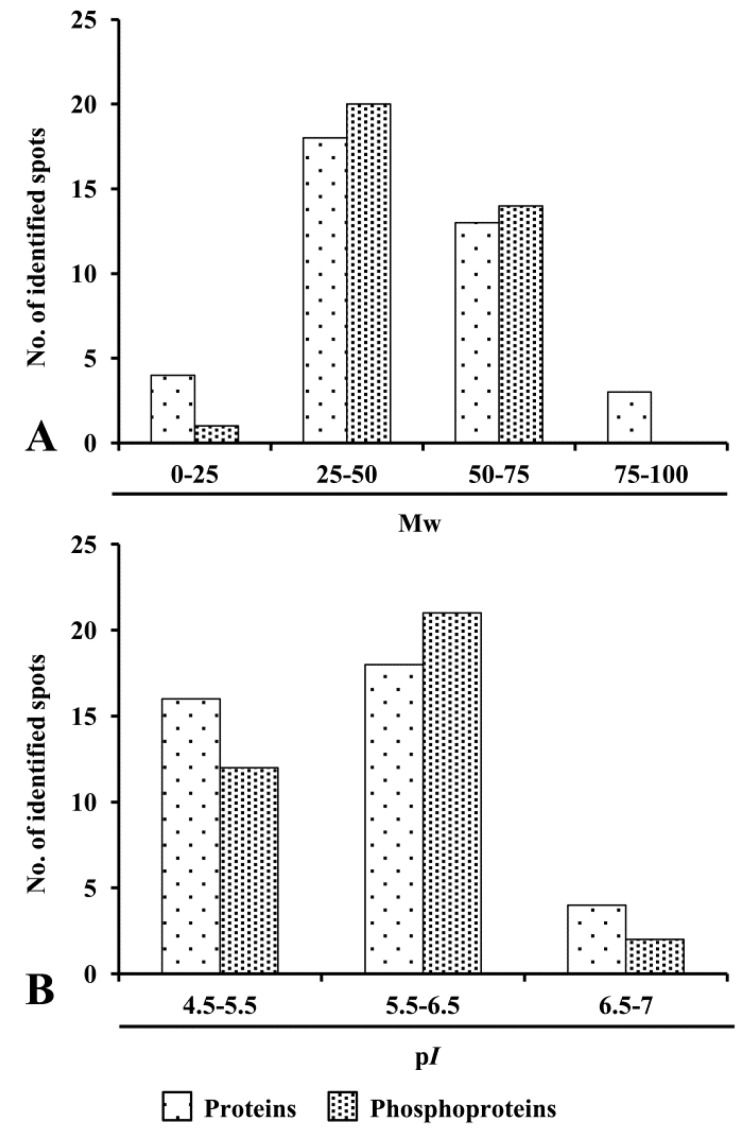
Physicochemical characteristics of proteins and phosphoproteins. Distribution of proteins and phosphoproteins based on (**A**) molecular weight (Mw) and (**B**) Isoelectric point (p*I*).

To examine putative characteristic features, the identified proteins and phosphoproteins were analyzed with respect to experimental molecular mass and p*I* distribution. 2-DE map of ECM was dominated by proteins having molecular weight of 0–50 kDa (60%) followed by 50–75 kDa and 75–100 kDa (Figure 3A). The majority of proteins exhibited a distribution pattern ranged from p*I* 5.5–6.5 (65%) followed by p*I* 4.5–5.5 (Figure 3B). Furthermore, physicochemical characteristics revealed that phosphoproteins showed predominance in molecular weight, ranging from 25–50 kDa and p*I* 5.5–6.5.

### 3.4. Functional Categorization of ECM Proteins

**Figure 4:**
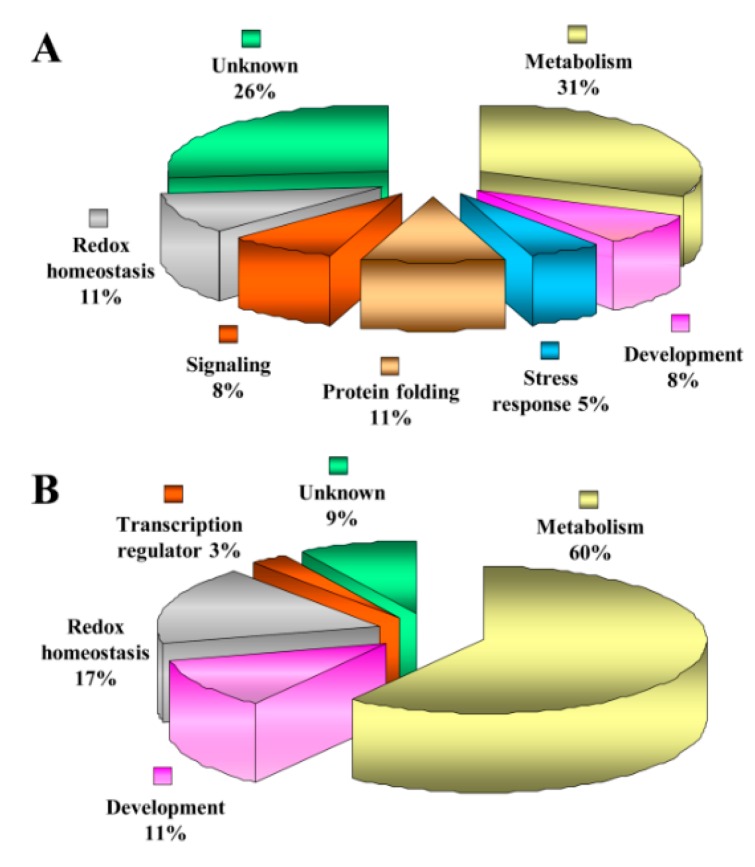
Functional cataloguing of identified spots. (**A**) Proteins and (**B**) phosphoproteins in ECM of potato. Putative functions were assigned to each of the candidates using protein function database.

To comprehend the function of the ECM proteins, the identified proteins were sorted into seven categories, as shown in Figure 4A. However, the classification of proteins is only tentative, since the biological function of many proteins identified has not yet been established experimentally. About 26% of the identified proteins were grouped under the unknown category, since no information as to their potential function in the organelle was available. Domain analysis of unknown proteins revealed that HAD-like domain, NAD(P)-binding domain, inorganic pyrophosphatase were identified in ECM proteome (Table S3). Proteins involved in metabolism (32%) were most abundant in our study. StEP 529 and 675 were identified to be alpha-1,4-glucanprotein synthase (UDP-forming). It is an ECM remodeler that is involved in architecture reformation and restructuring. Two isoforms of ErbB-3 binding protein (EBP1), StEP 1005 and 1213, were also present in the potato ECM proteome. EBP1 is known to function as a signal component in wall hydration [41]. 5-methyltetrahydropteroyltriglutamate homocysteine (StEP 1003) was included in this category, as it is a structure reorganizer for carbohydrate biosynthesis in ECM. The second largest category comprised proteins involved in redox homeostasis (11%). Glutathione recycle pathway enzymes were the predominant proteins in this category. These proteins contain glutathione degrading and forming enzymes. ROS degrading enzymes like peroxiredoxin (StEP 765) and thioredoxin peroxidase (StEP 615) represent the other set of ECM proteins in this category. Another important category of proteins identified are presumably involved in protein folding (10%) mechanism. Leucine aminopeptidase 2 (StEP 1024) is an amino acid dependent peptidase involved in proteolysis and peptidolysis. Interestingly, StEP 405 and 567, a chaperonin 60 protein identified in the potato ECM proteome is important for higher order protein organization and the regulation of the protein folding mechanism. Proteins involved in signaling are important attributes in the ECM proteome. In our study, three of the total identified proteins belonged to the signaling and development category. G-protein signaling components identified in this study might play a pivotal role in ECM organization and restructuring. Germin (StEP 1122 and 1333) were found to be a part of the ECM proteome of potato and might play a role in cell wall development. The stress-related proteins account for 5% of the total ECM proteome of potato that include chloroplast drought-induced stress protein of 32 kDa (CDSP32) protein (StEP 962 and 965). The presence of stress-related proteins in ECM has often been related to the response to different abiotic and biotic stresses. The subcellular localization of the identified proteins was also predicted by various subcellular prediction programs. Out of 38 proteins, 15 were predicted to be cell wall or ECM localized (Table S4).

### 3.5. Functional Categorization of ECM Phosphoproteins

Out of 35 phosphoproteins identified, we were able to assign functions to 32 ECM phosphoproteins, whereas three proteins belonged to unknown category. Domain analysis of these proteins was done using the InterPro database. The major domains were ubiquitin-conjugating enzyme and thioredoxin-like fold (Table S5). The phosphoproteins were classified into five different functional classes, as depicted in Figure 4B. Most phosphoproteins categorized under metabolism (60%) are characteristic cell wall proteins with distinct functions.  Approximately 17% of ECM phosphoproteins were related to redox homeostasis. The phosphorylation and dephosphorylation are mirror images and regulated by protein selectivity and activity. ROS degradation and regeneration are also known to be regulated by phosphorylation events. Detoxifying enzymes like superoxide dismutase (StEPP 24, 67, 111, and 225) identified in this data might be involved in ECM cross-linking. Development-related (11%) candidates covered proteins, such as germin-like protein, a known cell wall protein with multiple functions, which was also identified in the ECM phosphoproteome. StEPP 136 is a eukaryotic translation elongation factor, which does not possess any feature of ECM proteins. They are known to be related to transcriptional regulation (3%). Though, there is no direct evidence for a role of this protein in phosphorylated form in the ECM, but it might regulate accumulation of many ECM proteins. The subcellular localization of the identified phosphoproteins was predicted by various subcellular predictions and 10 were found to be canonical cell wall or ECM-localized proteins (Table S4). The sites of phosphorylation were predicted using Netphos (Centre for Biological Sequence Analysis, Lyngby, Denmark, version 2.0), Kinase Phos 2.0 (Institute of Bioinformatics, Taiwan, China, version 2.0), pkaPS prediction (Research Institute of Molecular Pathology, Vienna, Austria), and Phospho motif finder programs (John Hopkins University, MD, USA and Institute of Bioinformatics, Taiwan, China). These results revealed the highest possibility of phosphorylation on serine residues followed by threonine and tyrosine (Table S6).

**Figure 5:**
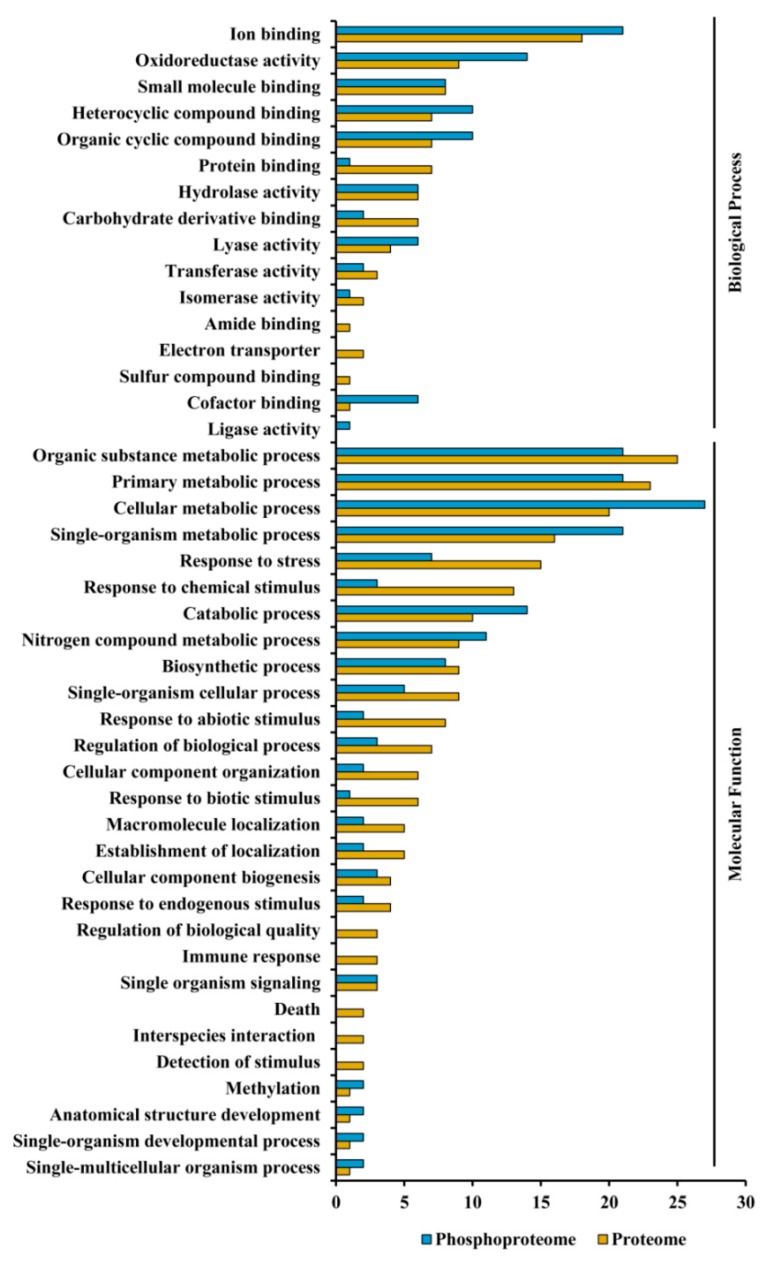
Functional classification of the identified ECM proteins and phosphoproteins. The non-redundant set of proteins was catalogued according to their gene ontology using Blast2GO program for predicted biological process and molecular function.

Further, Blast2GO analysis of the identified proteins and phosphoproteins revealed a wide range of biological processes and molecular functions, which can be classified into 16 and 25 categories, respectively. In terms of the number of proteins and phosphoproteins, the largest group within molecular function was found to be metabolic process (22.63% and 23.25%, respectively), followed by cellular process (19.25% and 14.28%, respectively) and response to stimulus (18.70% and 19.15%, respectively). The biological processes of these proteins mainly belonged to catalytic activity (53.23% and 48.21%, respectively), ion binding (18.22% and 12.25%, respectively) and structural molecule activity (5.23% and 4.11%, respectively) (see Figure 5).

### 3.6. Identification of Non-Canonical Proteins and Phosphoproteins in ECM

More than 6% of potato ECM proteins and 4% phosphoproteins were found to be noncanonical. These include metabolism associated proteins and phosphoproteins, like dehydratase (DH), putative beta-hydroxyacyl-ACP dehydratase (StEP 893), and glutamate dehydrogenase (StEP 1418), which may have a role in cell wall hydration and acidification [42]. The non-canonical stress-responsive plant protein, CDSP32 (StEP 962 and 965) is known to translocate to the cytosol upon perception of environmental cues [43]. In this study, G- protein signaling associated protein (StEP 1417) was, for the first time, found to be localized in the cell wall, thereby implying that signaling cascade may be initiated in the extra cellular matrix and culminate in the endosome or cytosol [44]. The presence of phosphorylated patatins (StEPP 38 and 80) in the ECM is significant with reference to its function towards nutritional status and stress response [45]. Balance between the cell growth, acidification, and hydration is crucial for maintenance of cell wall architecture. This balance mediated by non-cannonical proteoforms may be important to regulate cell wall homeostasis.

### 3.7. Cellular ECM Proteome and Phosphoproteome Network

**Figure 6:**
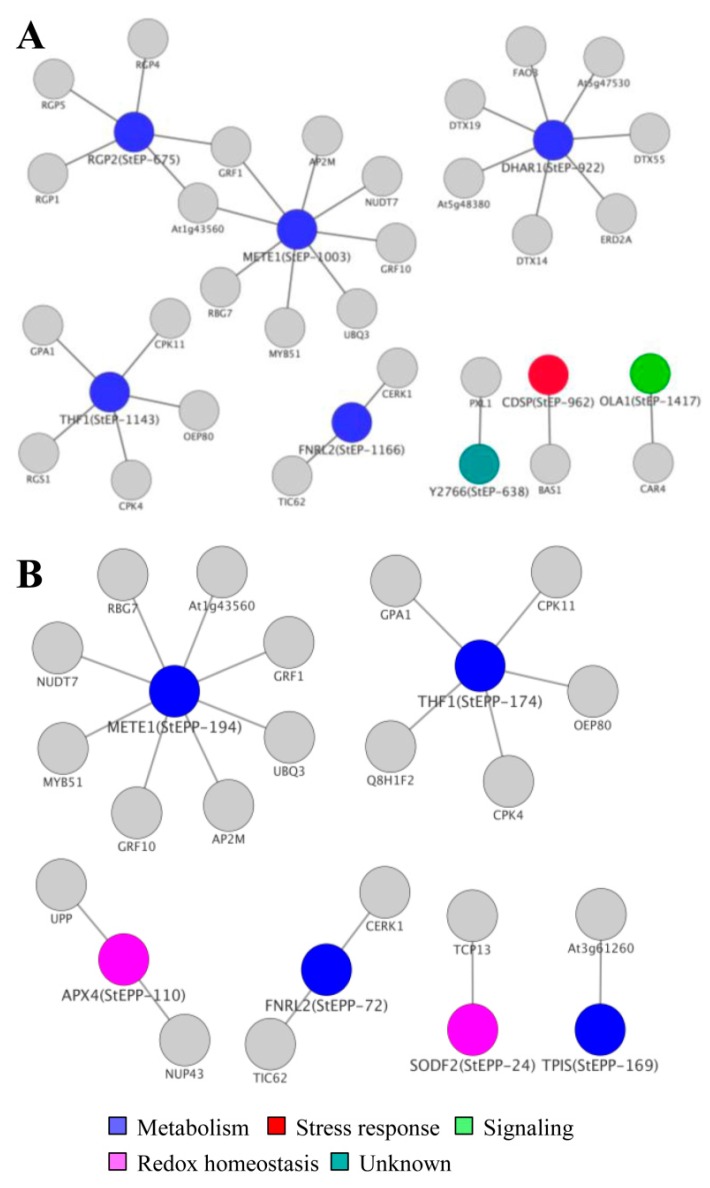
Protein-protein interaction network. (**A**) Proteins and (**B**) phosphoproteins. A spoke network was interconnected by querying PPI databases with all identified proteins and phosphoproteins for experimentally-determined interactions, as listed in manually curated databases. Nodes represent genes and edges represent interactions between genes. Node colors represent functional categories.

To understand the biological function and properties of ECM in depth, we next constructed the ECM proteoform networks delineating the alterations at proteome and phosphoproteome level. Complementary characteristics of the protein and phosphoprotein networks were categorized based on the functional attributes of the organelle in question. Our data suggests pathways related to mechanics and cross-linking polymer formation with tensile strength of ECM in the protein network (Figure 6A and Table S7). Three major groups were identified, of which group 1 (mechanics and tensile properties) mapped to proteins involved in pectin, lignin, and cellulose biosynthesis, including α-1, 4-glucan protein synthase (StEP 675), 5-methyltetrahydropterotriglutamate homocysteinase (StEP 1003) and dehydroascorbate reductase (StEP 922 and 1137). Group 2 comprised of three candidate proteins related to wall growth, signaling, and semantics. The identification of GTP binding proteins (StEP 1417), ferredoxin oxidoreductase (StEP 1166) and protein thylakoid formation (StEP 1143), indicate the role of signal components in ECM function and processes. Moreover, Group 3 consists of proteins associated with wall stress represented by CDSP 32 proteins (StEP 962 and 965) provided insight into the diversity of information afforded by network analysis. These data showed that ECM regulates the translational network essential for signal perception and cell architecture.

Next, we examined the phoshoprotein relays and interactors in the network to elucidate role of post translational modifications in the ECM (Figure 6B and Table S7). The wall pH and ROS formation is greatly affected by phosphoprotein’s non-stochiometric attributes. Our network was predominated with phosphoproteins related to redox homeostasis consisted of ascorbate peroxidase (StEPP 110 and 171), superoxide dismutase (StEPP 24, 67, 111, and 225), and ferredoxin reductase (StEPP 43, 72, 122, and 179) suggesting that these interactors might regulate physicochemical properties of the ECM. Furthermore, phosphoproteins associated with wall metabolism like triose phosphate isomerase (StEPP 169) and methionine synthase (StEPP 135 and 194) represent key components which are known to affect carbohydrate and amino acid metabolism [46]. Altogether, this data demonstrate that protein and phosphoprotein signatures can functionally link translational changes to organelle function.

## 4. Discussion

**Figure 7:**
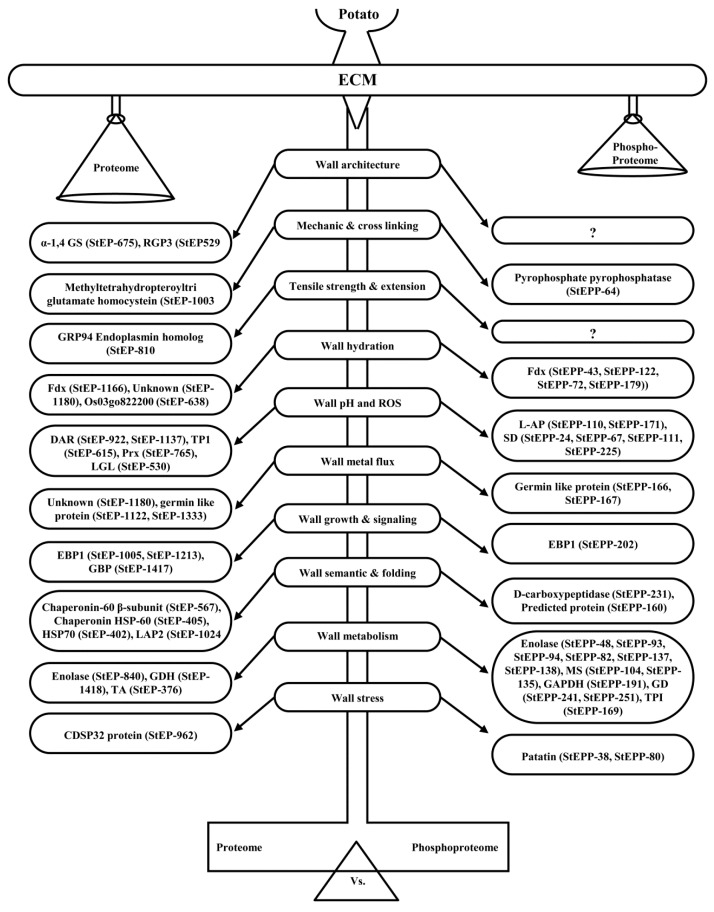
Model depicting diverse pathways putatively functional in the ECM of potato. Proteins and phosphoproteins identified in this study are referred to as StEP and StEPP, respectively. α-1,4 GS, α-1,4 Glucan synthase; RGP3, Reversibly glycosylated polypeptide 3; Fdx, Ferredoxin-NADP(H) oxidoreductase; DAR, Dehydroascorbate reductase-like protein; TPI, Triose phosphate isomerase; Prx, peroxiredoxin; LGL, Lactoylglutathione lyase; LAP2, Leucine aminopeptidase 2; GDH, Glutamate dehydrogenase; TA, Transaldolase; L-AP, L-ascorbate peroxidase; SD, Superoxide dismutase; MS, Methionine synthase; GAPDH, Glyceraldehyde 3-phosphate dehydrogenase.

This study is directed towards the systematic analyses of ECM proteoforms in a tuber crop, potato. A model illustrating the structural and functional network associated with cell wall integrity and dynamics is depicted in Figure 7. The structural network comprises the proteins and phosphoproteins known to participate in cell wall organization and remodeling.

### 4.1. ECM Proteoform Homeostasis Regulates Wall Architecture and Mechanics

The cell wall integrity as well as plasticity is the concerted effort of homeostatic properties in the dynamic milieu of ECM proteins and phosphoproteins. Turgor-driven expansion is the consequence of equilibrium between wall relaxation and stiffening linked by a physicochemical feedback loop [47]. Our study identified a few proteins related to wall architecture and maintenance, such as alpha 1,4 glucan synthase (StEP 675), methyltetrahydro-pteroyl triglutamate homocysteinase (StEP 1003), and GRP endoplasmin homolog (StEP 810), suggesting their role in turgor-driven cell wall extensibility. It is known that cell expansion depends on the turgor pressure and yield threshold of the wall in *Arabidopsis* [48]. Rheological properties of the wall determine rearrangement of wall polymers through hydrolysis, transglycosylation, phosphorylation, and disruption of hydrogen bonds to loosen the wall, making it extensible under turgor pressure [49], while structural carbohydrates modulate wall properties and strengthen the wall by rapid expansion [50]. We found that the ECM homeostasis is the interplay of diverse proteins, including dehydroascorbate reductase (StEP 922 and StEP 1137), thioredoxin peroxidase 1(StEP 615), peroxiredoxin (StEP 765), lactoglutathione lyase (StEP 530), and putative ferredoxin (StEP 1166), which might have a role in the release of reactive oxygen species (ROS), metal fluxes and pH balance. In addition, germin (StEP 1333) and 24 k germin-like protein (StEP 1122) are thought to promote wall rearrangement, thereby sustaining the structural framework.

Furthermore, we have reported the presence of ECM phosphoproteins controlling cell wall integrity, metal flux, and redox homeostasis. Phosphorylation of germin (StEPP 166 and 167) might control polymer length and also contribute to promote wall extensibility [51]. Phosphoprotein, pyrophosphatase (StEPP 64), identified in this study reflect that the posttranslational modification controls physicochemical parameter and reflect changes in wall mechanics. Phosphorylation of L-ascorbate peroxidase (StEPP 171 and 1101) and superoxide dismutase (StEPP 24, 67, 111, and 225) had been reported to influence phase shift of the pH and ROS oscillations [52].

### 4.2. ECM Proteoforms Mediate Semantics, Wall Signaling, and Metabolism

The interconnected network of ECM proteins and phosphoproteins participate in various biological processes, including signal perception, transduction, cellular transport, and metabolism. Interestingly, we identified EBP 1 associated with sensing mechanism and transduction in both non-phosphorylated (StEP 1005 and 1213) and phosphorylated forms (StEPP 202). Another signaling component, GTP binding protein (StEP 1417), had been reported to reflect plasticity of the wall deposition patterns required for the generation of complex cell shapes in higher plants [53]. There was a clear upsurge in the identification of molecular chaperones and heat shock proteins in potato ECM. This group of proteins is important for maintaining homeostasis by regulating protein folding, assembly, translocation, and degradation [54]. In addition to protein folding proteins, our analysis also identified peptidase-like leucine aminopeptidase (StEP 1024), shown to be involved in the degradation of proteins and phosphoprotein with high entropy [55]. Regulation of the cell wall metabolism is pivotal for controlling plant growth and development. We found enolase (StEP 840), glutamate dehydrogenase (StEP 1418) and trans-ketolase (StEP 376) associated with wall deposition and biosynthesis. Phosphorylation of metabolic proteins like enolase (StEPP 48, 82, 93, 137, and 138), methionine synthase (StEPP 104 and 135), glyceraldehyde 3 phosphate dehydrogenase (StEPP 191), glutamate dehydrogenase (StEPP 241 and 251), and triose phosphate isomerase (StEPP 169) might play major roles in maintaining protein-matrix ratio. Altogether, our study provides a model for cell wall integrity, dynamics, and intercellular communication.

### 4.3. Comparison of Extracellular Matrix Proteomes

Extracellular matrix proteins show diverse composition in different families of plant kingdom. To examine the composition and evolution of ECM proteomes at organismal level, we compared the representative ECM proteomes of a Solanaceae (potato (this study) and tobacco) and Brassicaceae (*Arabidopsis* (suspension culture and hypocotyl) and *Brassica* (root)) [56,57,58]. We found a great level of divergence in the protein classes among these organisms. To our surprise, only one ECM protein (peroxiredoxin) was found to be common in all the organisms studied thus far. It may be speculated that ECM protein diversity in Solanaceae and Brassicaceae is interrelated with the ecological niche of the corresponding organism. Despite evolutionary variation, our study also revealed similarities in few essential functional protein classes. The presence of orthologues of redox homeostasis related protein, such as peroxidases reconfirms the role of homeostasis regulator against various stress factors irrespective of the organism studied. Further, presence of orthologs of families of proteases, peptidases, and heat shock proteins in ECM of *Arabidopsis*, potato, and *Brassica* entails a common layout of the protein folding and degradation machinery in the outermost layer of plants. The presence of the candidate protein stress marker, like germin in potato and *Arabidopsis*, may be attributed to their adaptability to a high degree of environmental stresses. Proteins involved in cellular metabolism, such as α-1-4-glucan synthase and 1,3 glycosyl transferases were found to be conserved in *Arabidopsis*, while enolase was found to be present exclusively in potato. This observation clearly suggests that different proteins might have similar function in ECM depending on species. Furthermore, it implies that difference in response lies in the regulation and functional modularity of protein involved in different biological processes.

## Figures and Tables

**Table 1:** List of identified ECM proteins of potato by MS/MS analysis.

Functional Category	Spot ID ^a.^	Protein Name	Species	Protein ID ^b^	Score	NP ^c^	% coverage	Thr. Mw/p*I*	Exp. Mw/p*I*
Metabolism	StEP-840	Enolase	*Lycopersicon esculentum*	119354	88	3	10	47.768/5.68	60.334/5.49
	StEP-1003	5-ethyltetrahyd ropteroyltriglutamate-homocysteine	*Solanum tuberosum*	XP_002328083.1	198	5	6	84.54/6.26	53.065/5.82
	StEP-1166	Ferredoxin-NADP(H) oxidoreductase	*Oryza sativa (*japonica cultivar-group*)*	41052915	57	2	5	40.638/7.98	40.844/6.22
	StEP-1418	Glutamate dehydrogenase	*Lycopersicon esculentum*	12229803	441	9	26	44.78/6.68	53.599/6.94
	StEP-675	Alpha-1,4-glucan-protein synthase	*Solanum tuberosum*	34582499	216	8	22	41.57/5.71	51.107/5.29
	StEP-529	RGP3 (Reversibly glycosylated polypeptide 3)	*Arabidopsis thaliana*	30680679	69	2	4	41.25/5.39	50.666/4.94
	StEP-376	Transaldolase	Unknown species	85112172	78	2	6	35.20/5.38	45.309/4.62
	StEP-1143	Protein thylakoid formation1	*Solanum tuberosum*	75140959	140	5	20	33.34/8.69	28.646/5.89
	StEP-1213	ErbB-3 binding protein1	*Solanum tuberosum*	116292768	320	7	21	42.79/6.25	58.028/6.37
	StEP-1005	ErbB-3 binding protein1	*Solanum tuberosum*	116292768	147	5	13	42.79/6.25	58.891/5.87
	StEP-893	DH putative beta-hydroxyacyl-ACP dehydratase	*Capsicum annuum*	193290688	138	4	14	23.966/9.35	22.904/5.64
Development	StEP-1333	Germin-like protein 1	*Nicotiana tabacum*	1169944	58	1	4	21.98/6.27	26.698/6.75
	StEP-1122	24K germin like protein	*Nicotiana tabacum*	31711507	67	2	15	21.954/5.82	26.698/6.75
	StEP-656	Chain A, The Crystal Structure Of Semet Patatin	*Solanum tuberosum*	31615943	72	2	4	40.93/5.17	39.366/5.25
Stress Response	StEP-962	Chloroplast Drought-induced Stress Protein of 32 kDa	*Solanum tuberosum*	2582822	221	7	20	33.436/8.07	37.4815.64
	StEP-965	Chloroplast Drought-induced Stress Protein of 32 kDa	*Solanum tuberosum*	2582822	179	7	20	33.436/8,07	37.525/5.83
Protein Folding	StEP-567	Chaperonin-60 beta subunit	*Solanum tuberosum*	1762130	275	7	17	62.98/5.72	76.934/4.85
	StEP-405	Chaperonin hsp60	*Arabidopsis thaliana*	16221	279	7	12	61.31/5.66	70.819/5.12
	StEP-402	Heat shock protein 70	*Nicotiana tabacum*	30025966	457	8	13	70.83/5.17	76.624/5.06
	StEP-1024	Leucine aminopeptidase 2	*Lycopersicon esculentum*	2492530	191	5	11	59.512/8.18	69.045/5.67
Signaling	StEP-1417	GTP-binding protein	*Capsicum annuum*	7643796	433	12	38	44.37/6.45	57.878/6.88
	StEP-1001	Glucose-regulated protein 78	*Lycopersicon esculentum*	170386	177	5	12	41.15/8.51	55.669/5.78
	StEP-810	Endoplasmin homolog	*Hordeum vulgare*	544242	52	1	1	92.859/4.86	41.936/5.47
Redox Homeostasis	StEP-1137	Dehydroascorbate reductase-like protein	*Solanum tuberosum*	76160951	62	2	9	23.425/6.09	28.647/5.99
	StEP-922	Dehydroascorbate reductase	*Lycopersicon esculentum*	66475036	61	2	9	23.523/6.32	28.508/5.79
	StEP-615	Thioredoxin peroxidase 1	*Lycopersicon esculentum*	30841938	57	2	11	17.426/5.18	20.169/5.68
	StEP-765	Peroxiredoxin	*Ricinus communis*	223543823	55	1	5	21.82/8.74	22.953/5.42
	StEP-530	Lactoylglutathione lyase	*Ricinus communis*	223542315	249	4	16	31.52/7.63	42.983/4.98
Unknown	StEP-848	Unknown protein 18	*Pseudotsuga menziesii*	205830697	63	1	91	1.393/5.80	70.364/5.37
	StEP-853	Unknown protein 18	*Pseudotsuga menziesii*	205830697	50	2	100	1.393/5.80	79.178/5.508
	StEP-1004	Unknown protein 18	*Pseudotsuga menziesii*	205830697	83	2	100	1.39/5.80	58.661/5.83
	StEP-352	Unknown protein 18	*Pseudotsuga menziesii*	205830697	99	2	100	1.39/5.80	40.823/4.82
	StEP-879	Unknown protein 19	*Pseudotsuga menziesii*	205830698	54	1	91	1.393/5.80	13.226/5.69
	StEP-1180	Unknown	*Solanum tuberosum*	77745483	281	7	20	37.64/7.70	45.963/6.01
	StEP-354	Unknown	*Populus trichocarpa*	118486367	71	3	8	44.55/8.48	41.326/4.72
	StEP-963	Hypothetical protein	*Pseudotsuga menziesii*	83649448	55	2	2	36.547/6.47	37.799/5.78
	StEP-346	Predicted protein	*Populus trichocarpa*	224103823	113	2	7	33.55/6.85	38.700/5.02
	StEP-638	Os03g0822200	*Oryza sativa (*japonica cultivar-group*)*	115456265	57	1	6	27.89/6.34	33.319/5.17

^a^ Spot number as marked on the ECM proteome 2-D gel image Figure 2A. The spot numbers were designated as StEP, where St indicates the organism *Solanum tuberosum*, EP denotes the Extracellular Matrix Proteome. ^b^ Protein identification number as in gene bank. ^c^ Number of peptides.

**Table 2:** List of identified ECM phosphoproteins of potato by MS/MS analysis.

Functional Category	Spot ID ^a.^	Protein Name	Species	Protein ID ^b^	Score	NP ^c^	% coverage	Thr. Mw/p*I*	Exp. Mw/p*I*
Metabolism	StEPP-48	Enolase	*Lycopersicon esculentum*	119354	176	5	15	47.76/ 5.68	61.074/5.05
	StEPP- 93	Enolase	*Lycopersicon esculentum*	119354	812	16	48	47.768/5.68	60.703/5.18
	StEPP-138	Enolase	*Lycopersicon esculentum*	119354	522	12	38	47.76/5.68	55.289/5.47
	StEPP-137	Enolase	*Lycopersicon esculentum*	119354	818	19	51	47.76/5.68	55.058/5.64
	StEPP-82	Enolase	*Lycopersicon esculentum*	3023685	104	2	6	47.56/5.41	53.226/5.53
	StEPP-94	Enolase-like	*Solanum tuberosum*	82623425	487	12	37	48.002/7.53	60.888/5.32
	StEPP-194	Methionine synthase	*Solanum tuberosum*	8439545	557	12	18	84.61/5.93	56.404/5.84
	StEPP-135	Cobalamine-independent methionine synthase	*Solenostemon scutellarioides*	974782	161	3	4	86.71/6.17	51.932/5.49
	StEPP-72	Ferredoxin-NADP reductase	*Nicotiana tabacum*	3913651	216	9	29	40.41/8.37	41.686/5.28
	StEPP-179	Ferredoxin-NADP reductase	*Nicotiana tabacum*	3913651	584	14	48	40.419/8.37	41.408/5.79
	StEPP-43	Ferredoxin-NADP reductase	*Nicotiana tabacum*	3913651	55	3	11	40.419/8.37	59.082/5.21
	StEPP-122	Ferredoxin-NADP reductase	*Nicotiana tabacum*	3913651	238	8	18	40.419/8.37	41.962/5.54
	StEPP-191	Glyceraldehyde 3-phosphate dehydrogenase	*Solanum tuberosum*	22094840	319	7	31	26.62/6.34	48.900/6.38
	StEPP-241	Glutamate dehydrogenase	*Lycopersicon esculentum*	12229803	688	15	39	44.78/6.68	54.134/6.79
	StEPP-251	Glutamate dehydrogenase	*Lycopersicon esculentum*	12229803	99	3	8	44.785/6.68	58.542/6.61
	StEPP-169	Triose phosphate isomerase cytosolic isoform-like	*Solanum tuberosum*	77745458	171	6	33	26.99/5.73	27.782/5.85
	StEPP-233	Succinic semialdehyde reductase isoform1	*Solanum lycopersicum*	171854589	247	6	31	30.428/6.60	35.085/6.44
	StEPP-174	Protein Thylakoid formation1	*Solanum tuberosum*	75140959	74	2	9	33.34/8.69	29.287/5.89
	StEPP-64	Soluble inorganic pyrophosphatase	*Arabidopsis thaliana*	2500047	163	5	28	24.24/5.59	28.967/5.28
	StEPP-202	ErbB-3 binding protein	*Solanum tuberosum*	116292768	346	9	27	42.79/6.25	59.333/5.94
	StEPP-231	Endolysin	*Vitis vinifera*	220928911	57	1	3	26.76/8.90	41.559/6.13
Development	StEPP-167	Germin-like protein	*Capsicum chinense*	123965222	51	2	7	21.03/7.74	27.647/5.77
	StEPP-166	Germin-like protein	*Capsicum chinense*	123965222	101	2	7	21.03/7.74	26.158/5.75
	StEPP-80	Patatin	*Solanum tuberosum*	129640	55	1	3	42.463/5.34	51.770/5.47
	StEPP-38	Patatin-11	*Solanum tuberosum*	122201875	270	6	18	42.47/5.58	51.132/5.23
Redox Homeostasis	StEPP-110	Putative l-ascorbate peroxidase	*Lycopersicon esculentum*	11387206	467	9	21	37.72/8.27	32.795/5.94
	StEPP-171	Putative l-ascorbate peroxidase	*Lycopersicon esculentum*	11387206	295	8	34	37.72/8.27	31.295/5.96
	StEPP-111	Iron superoxide dismutase	*Solanum tuberosum*	170675264	114	3	25	16.246/5.69	27.594/5.67
	StEPP-225	Iron superoxide dismutase	*Solanum tuberosum*	170675264	64	2	12	16.24/5.69	27.375/6.03
	StEPP-24	Superoxide dismutase	*Solanum lycopersicum*	33413303	120	3	15	27.893/6.60	27.456/5.19
	StEPP-67	Iron superoxide dismutase	*Solanum tuberosum*	27543371	255	6	33	21.31/5.87	26.943/5.27
Transcriptiom regulator	StEPP-136	Eukaryotic translation elongation factor	*Ricinus communis*	223547312	314	7	9	93.99/5.87	44.550/5.58
Unknown	StEPP-221	Unknown	*Solanum tuberosum*	81076617	72	4	18	24.95/7.77	28.551/6.12
	StEPP-180	Unknown	*Medicago truncatula*	217072754	54	2	7	27.84/5.51	33.564/5.88
	StEPP-160	Predicted protein	*Populus trichocarpa*	224091917	86	3	26	16.610/6.20	20.264/6.25

^a^ Spot number as marked on the ECM phosphoproteome 2-D gel image (Figure 2B). The spot numbers were designated as StEPP, where St indicates the organism *Solanum tuberosum*, EPP denotes the extracellular matrix phosphoproteome. ^b^ Protein identification number as in gene bank. ^c^ Number of peptides.

## Supplementary Materials

The following are available online at www.mdpi.com/2227-7382/4/3/20/s1, Figure S1: Scatter plots displaying a correlation coefficient of variation above 0.8 among the three replicates, Figure S2: 2-DE reference maps of the potato ECM proteins and phosphoproteins zoomed onto pH 4–7 13 cm. (**A**) ECM proteins, (**B**) ECM phosphoproteins. The second dimension was performed on 12.5% (*w*/*v*) SDS-PAGE, Figure S3: Reproducible 2-DE images of ECM proteome and phosphoproteome. (**A**) Proteins, (**B**) Phosphoproteins, zoomed onto 24 cm IPG strip (pH 4–7) and second dimension was performed on 12.5% (*w*/*v*) SDS-PAGE. The spot numbers marked in red arrows on three replicate gels correlate with the protein and phosphoprotein identifications as listed in Table 1 and Table 2, Figure S4: Venn diagram depicting overlaps of proteome and phosphoproteome, Table S1: The MS/MS detail of the identified protein spots by LC-MS/MS and The MS/MS detail of the identified phosphoprotein spots by LC-MS/MS, Table S2: Fragment spectra of the proteins identified with a single peptide and Fragment spectra of the phosphoproteins identified with a single peptide, Table S3: Domain analysis of ECM proteins, Table S4: Localization of ECM proteins and phosphoproteins, Table S5: Domain analysis of ECM phosphoproteins, Table S6: Phospho-site identification of ECM phosphoprotiens in potato, Table S7: List of interacting partners in phsophoprotein network depicted in Figure 6.

## Abbreviations

The following abbreviations are used in this manuscript

DTT: Dithiothreitol
PMSF: Phenylmethylsulfonyl fluoride
ACN: Acetonitrile
SDS: Sodium dodecyl sulfate
ATP: Adenosine Triphosphate
SDS PAGE: Sodium dodecyl sulfate polyacrylamide gel electrophoresis
LC-MS/MS: Liquid Chromatography-tandem mass spectrometry
