# Supplementary material for: Extracellular Matrix Proteome and Phosphoproteome of Potato Reveals Functionally Distinct and Diverse Canonical and Non-Canonical Proteoforms
Source: Proteomes. 2016 Jun 24;4(3):20. doi: 10.3390/proteomes4030020 (PMC5217357; doi:10.3390/proteomes4030020)
Supplement: Supplementary file 1 [file proteomes-04-00020-s001.zip › proteomes-04-00020-supplementary/proteomes-04-00020-supplmentary.pdf]

Extracellular Matrix Proteome and Phosphoproteome of Potato Reveals Functionally Distinct and Diverse Canonical and Non-Canonical Proteoforms

Eman Elagamey, Kanika Narula, Arunima Sinha, Pooja Rani Aggarwal, Sudip Ghosh, Niranjan Chakraborty and Subhra Chakraborty

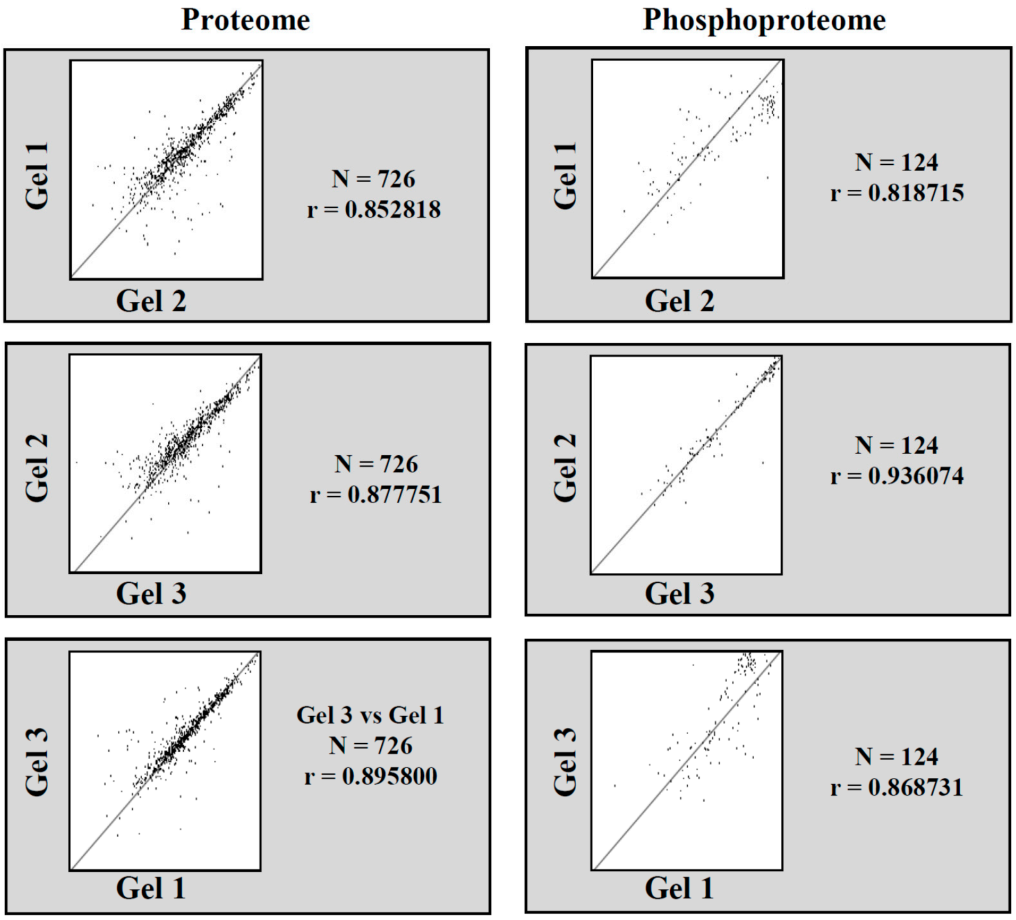

Figure S1. Scatter plots displaying a correlation coefficient of variation above 0.8 among the three replicates.

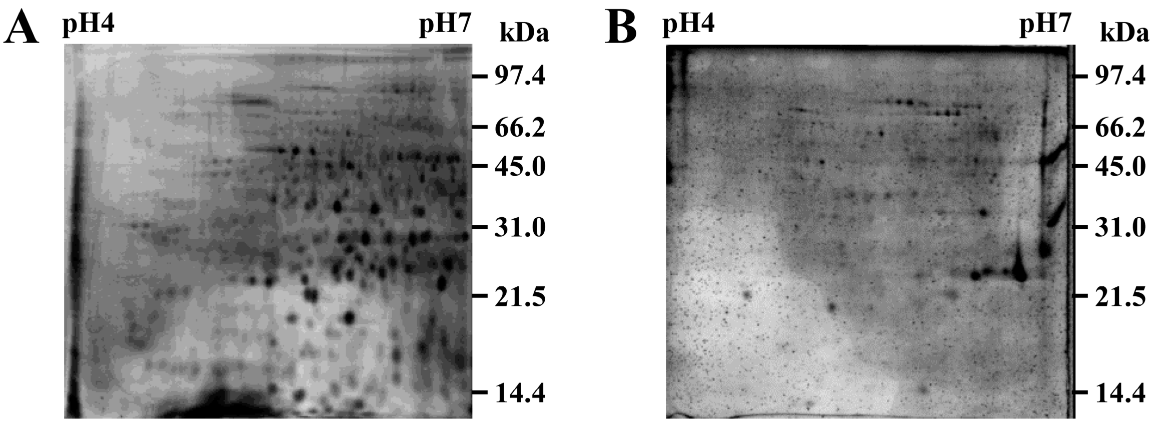

Figure S2. 2-DE reference maps of the potato ECM proteins and phosphoproteins zoomed onto pH 4-7 13 cm. (**A**) ECM proteins, (**B**) ECM phosphoproteins. The second dimension was performed on 12.5% (*w*/*v*) SDS-PAGE.

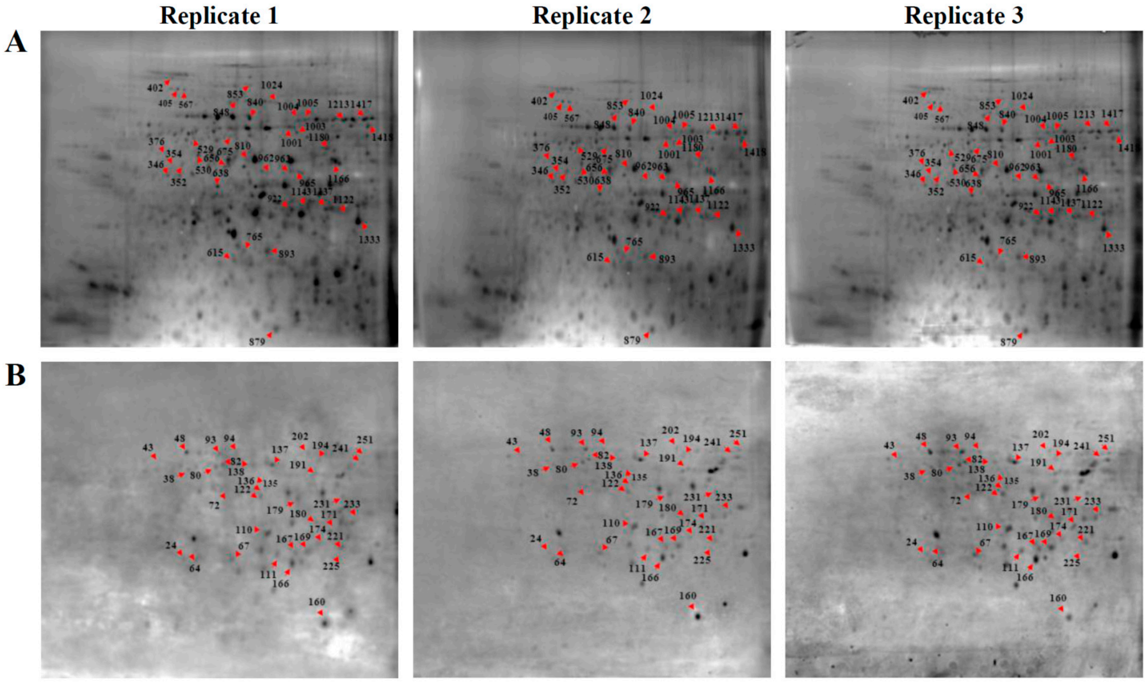

Figure S3. Reproducible 2-DE images of ECM proteome and phosphoproteome. (**A**) Proteins, (**B**) Phosphoproteins, zoomed onto 24 cm IPG strip (pH 4-7) and second dimension was performed on 12.5% (*w*/*v*) SDS-PAGE. The spot numbers marked in red arrows on three replicate gels correlate with the protein and phosphoprotein identifications as listed in Table 1 and Table 2.

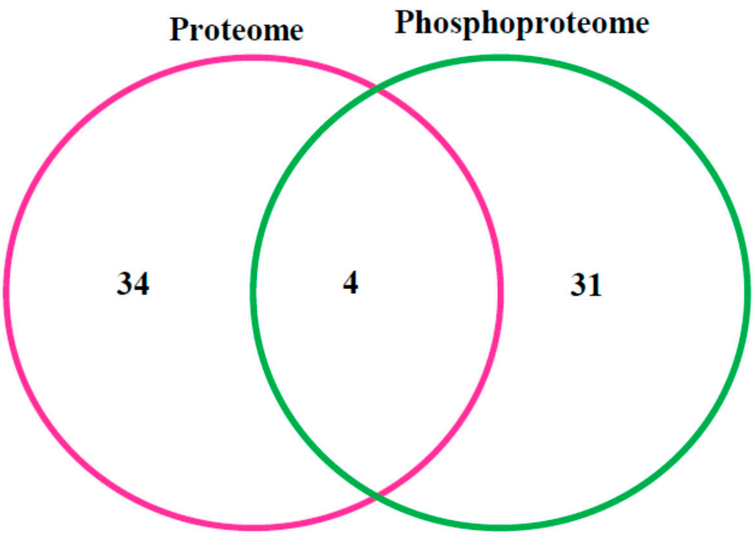

Figure S4. Venn diagram depicting overlaps of proteome and phosphoproteome.
