# Supplementary material for: Extracellular Matrix Proteome and Phosphoproteome of Potato Reveals Functionally Distinct and Diverse Canonical and Non-Canonical Proteoforms
Source: Proteomes. 2016 Jun 24;4(3):20. doi: 10.3390/proteomes4030020 (PMC5217357; doi:10.3390/proteomes4030020)
Supplement: Supplementary file 1 [file proteomes-04-00020-s001.zip › proteomes-04-00020-supplementary/Table S3.pdf]

Table S3. Domain analysis of ECM proteins.

Spot ID [a]	Protein Name	Protein ID [b]	Interpro/Pfam [c]	Domain Name [d]
StEP-354	Unknown	118486367	IPR023214	HAD-like domain
			IPR006357	HAD-superfamily hydrolase, subfamily IIA
			IPR023215	Nitrophenylphosphatase-like domain
			IPR006349	2-phosphoglycolate phosphatase, eukaryotic
StEP-346	Predicted protein	224103823	IPR008162	Inorganic pyrophosphatase
StEP-638	Os03g0822200	115456265	IPR016040	NAD(P)-binding domain

[a] Spot number as given on the 2-D gel images. The first letters (St) signify the source plant, *Solanum tuberosum*, followed by EP denotes the Extracellular Matrix Proteome. The numerals indicate the spot numbers corresponding to Figure 3A. [b] Protein identification number as in GenBank. [c] InterPro domain accession number.
