# Supplementary material for: Extracellular Matrix Proteome and Phosphoproteome of Potato Reveals Functionally Distinct and Diverse Canonical and Non-Canonical Proteoforms
Source: Proteomes. 2016 Jun 24;4(3):20. doi: 10.3390/proteomes4030020 (PMC5217357; doi:10.3390/proteomes4030020)
Supplement: Supplementary file 1 [file proteomes-04-00020-s001.zip › proteomes-04-00020-supplementary/Table S5.pdf]

**Table S5.** Domain analysis of ECM phosphoproteins.

| Spot ID [a] | Protein Name | Protein ID [b] | Interpro/Pfam [c] | Domain Name [d] |
|---|---|---|---|---|
| StEPP-221 | Unknown | 81076617 | IPR012336 | Thioredoxin-like fold |
| StEPP-160 | Predicted protein | 224091917 | IPR000608 | Ubiquitin-conjugating enzyme, E2 |
| | | | IPR016135 | Ubiquitin-conjugating enzyme/RWD-like |

[a] Spot number as given on the 2-D gel images. The first letters (St) signify the source plant, *Solanum tuberosum*, followed by EPP denotes the Extracellular Matrix Phosphoproteome. The numerals indicate the spot numbers corresponding to Figure 3B. [b] Protein identification number as in GenBank. [c] InterPro domain accession number. [d] For each of the proteins identified as "unknown functions" in Table 2, the InterPro site was queried for domains in SMART, Panther, and Pfam databases to identify functional domains of each protein. N/F, not found; N/A, not applicable.
